# Temporal trends in the birth rates and perinatal mortality of twins: A population-based study in China

**DOI:** 10.1371/journal.pone.0209962

**Published:** 2019-01-16

**Authors:** Changfei Deng, Li Dai, Ling Yi, Xiaohong Li, Kui Deng, Yi Mu, Ke Wang, Jing Tao, Qi Li, Liangzhi Xu

**Affiliations:** 1 National Center for Birth Defects Monitoring, West China Second University Hospital, Sichuan University, Chengdu, Sichuan, China; 2 Department of Obstetrics and Gynecology, West China Second University Hospital, Sichuan University, Chengdu, Sichuan, China; 3 Chinese Evidence-based Medicine Center, West China Hospital, Sichuan University, Chengdu, China; 4 Key Laboratory of Birth Defects and Related Diseases of Women and Children (Sichuan University), Ministry of Education, Chengdu, Sichuan, China; Public Library of Science, UNITED KINGDOM

## Abstract

**Objective:**

Until now, little was known about the epidemiological characteristics of twins in China due to a lack of reliable national data. In this study, we aimed to analyze temporal trends and perinatal mortality of twins from China.

**Methods:**

Data on twins between 2007 and 2014 were obtained from the China National Population-Based Birth Defects Surveillance System. Twin and singleton deliveries after at least 28 weeks of gestation were recruited and followed until postnatal day 42. Twinning rates were defined as the number of twin individuals per 1000 births(stillbirths and live births). The Weinberg’s differential method was utilized to estimate the number of monozygotic and dizygotic twins.

**Results:**

During 2007–2014, the twinning rate increased by 32.3% from 16.4 to 21.7 per 1000 total births with an average of 18.8‰. Among twins, both the perinatal mortality rate (26.1 per 1000 total births) and neonatal death rate (15.7 per 1000 live births) presented a downward tendency but remained at a high level. Large urban-rural and geographic disparities were identified in twinning rates, in perinatal and neonatal mortality, and in their temporal trends.

**Conclusions:**

The upward trend of twinning rates in China paired with the relatively high rates of perinatal and neonatal mortality among twins highlights the need for improved perinatal care in the light of socio-demographic differences.

## Introduction

Twins account for 0.5–4.0% of all births, with rates that vary across different ethnicities and countries [1–4]. Compared with singletons, twins experience a higher risk of adverse perinatal outcomes, including preterm delivery, low birth weight, and increased rates of perinatal and neonatal morbidity and mortality [5–11]. Furthermore, both twin pregnancies and twin individuals often require special prenatal and postnatal care. As a result, additional epidemiological data on the rates and clinical outcomes of twin pregnancies will have significant implications for the assessment of perinatal and neonatal health, as well as the allocation of healthcare resources.

Over the last three decades twinning rates have increased remarkably worldwide [12, 13], resulting in negative impacts on fetal, perinatal and neonatal mortality rates [5, 11, 14]. While twinning rates have been extensively studied in western countries, there is a paucity of reliable data available for such rates in China despite the fact that China accounts for one-fifth of the world’s total population. Using data for the years 2007–2014 from the China National Population-Based Birth Defects Surveillance System (NPBDSS) [15, 16], we aimed to identify trends in both birth rates and perinatal mortality for twins and identify socio-demographic disparities in twinning rates and related mortalities.

## Materials and methods

### Data source

Data for this analysis were obtained from the NPBDSS, a nationwide surveillance system covering 64 counties and districts in thirty provinces, municipalities or municipal districts directly under the central government. Fetuses and neonates born after at least 28 weeks of gestation born to women living in the surveillance areas for at least one year were recruited and followed until 42 days after birth. Data collected from this surveillance network include birth date, gender, parity, birth weight, outcome and birth defects diagnosed up to 42 days after birth. Data on twins and singletons who were born between 2007 and 2014 were used for the current analysis. Detailed information on data collection and quality control has been documented in other reports [15, 16].

### Epidemiological measurements and determinants

Twinning rate was defined as the number of twin individuals per 1000 total births (including both stillbirths and live births). The numbers of monozygotic and dizygotic twins were estimated by using Weinberg’s differential method [17]. Stillbirth and perinatal mortality rates were calculated as the number of stillbirths and perinatal deaths per 1000 total births, respectively. Early neonatal death and neonatal death rates were calculated as the number of corresponding deaths divided by the number of live births.

Other examined socio-demographic, maternal or infant risk factors included birth area (urban vs rural) [15, 16, 18], geographic region (eastern, central and western region in place of previous geo-economical classification of coastal, inner land and remote area) [16, 19], residence registration (local permanent and temporary household registration; individuals with temporary registration are categorized into floating population in China and usually have poor health care status and economic level) [20, 21], maternal age (<35 vs ≥ 35 years of age, younger vs older or advanced maternal age, AMA), parity (nulliparous vs parous women), ethnicity (depending on maternal ethnicity and combined in Han nationality, the main nationality in China, with the largest amount of the population, and minorities, with 55 Chinese minorities in total) and infant sex.

### Statistical analysis

The 95% confidence intervals (CIs) of the rates were calculated based on Poisson distribution. Pearson’s chi-square test was used to compare rates among groups, and linear chi-square test was employed to detect secular trends in rates. The statistical significance level for α was set at 0.05.

## Results

### Twinning rates

During the period from 2007 to 2014, a total of 49,028 twin individuals and 2,564,759 singleton births were recorded by the NPBDSS, yielding an overall rate of 18.8 per 1000 births (Table 1). Twinning rates were significantly higher in urban vs rural areas (20.4 vs 17.1), in eastern vs central and western regions (20.3 vs 17.3 and 17.4), for local vs temporary household registrations (19.2 vs 16.6), for female vs male twins (19.4 vs 18.2), in minorities vs Han nationality (19.6 vs 18.7), and for older (≥ 35 years) vs younger (<35 years) maternal ages (29.4 vs 17.9). Multivariable analysis further confirmed that the above-mentioned maternal and infant characteristics were in association with twinning rates (S1 Table).

**Table 1:** Twinning rates in Chinese population, 2007–2014.

Group	No of births (%)	No of twins (%)	Twin rate [‰,(95%CI)]
Birth area			
urban	1289729 (49.3)	26346 (53.7)	20.4 (20.2, 20.7)
rural	1324058 (50.7)	22682 (46.3)	17.1 (16.9, 17.4)
Geographic region			
eastern	1220917 (46.7)	24836 (50.7)	20.3 (20.1, 20.6)
central	744905 (28.5)	12912 (26.3)	17.3 (17.0, 17.6)
western	647965 (24.8)	11280 (23.0)	17.4 (17.1, 17.7)
Residence registration^#^			
local	2200124 (84.2)	42148 (86.0)	19.2 (19.0, 19.3)
temporal	413371 (15.8)	6876 (14.0)	16.6 (16.2, 17.0)
Gender*			
male	1393729 (53.3)	25369 (51.7)	18.2 (18.0, 18.4)
female	1219383 (46.7)	23641 (48.2)	19.4 (19.1, 19.6)
Ethnicity^ǂ^			
Han	2432343 (93.1)	45468 (92.7)	18.7 (18.5, 18.9)
minority	181437 (6.9)	3560 (7.3)	19.6 (19.0, 20.3)
Maternal age (yrs)^$^			
<35	2422349 (92.7)	43438 (88.6)	17.9 (17.8, 18.1)
≥35	188272 (7.2)	5534 (11.3)	29.4 (28.6, 30.2)
Parity^&^			
nulliparous	1862025 (71.2)	34980 (71.3)	18.8 (18.6, 19.0)
parous	750807 (28.7)	14028 (28.6)	18.7 (18.4, 19.0)
Total	2613787 (100)	49028 (100)	18.8 (18.6, 19.0)

The confidence interval of rate was calculated based on Poisson distribution.

^#^292 infants with unspecified maternal residence were excluded.

*675 births with unknown or unspecified gender were excluded.

^ǂ^7 births with unknown maternal ethnicity were excluded.

^$^3166 births with unknown maternal age were excluded.

^&^955 births with unknown parity were excluded.

Varied significantly by birth area(χ^2^ = 386.0, p<0.001), geographic region (χ^2^ = 311.9, p<0.001), Residence registration (χ^2^ = 120.360, p<0.001), gender(χ^2^ = 49.6, p<0.001), ethnicity (χ^2^ = 7.8, p<0.01), and maternal age(χ^2^ = 1079.4, p<0.001).

**Fig 1:**
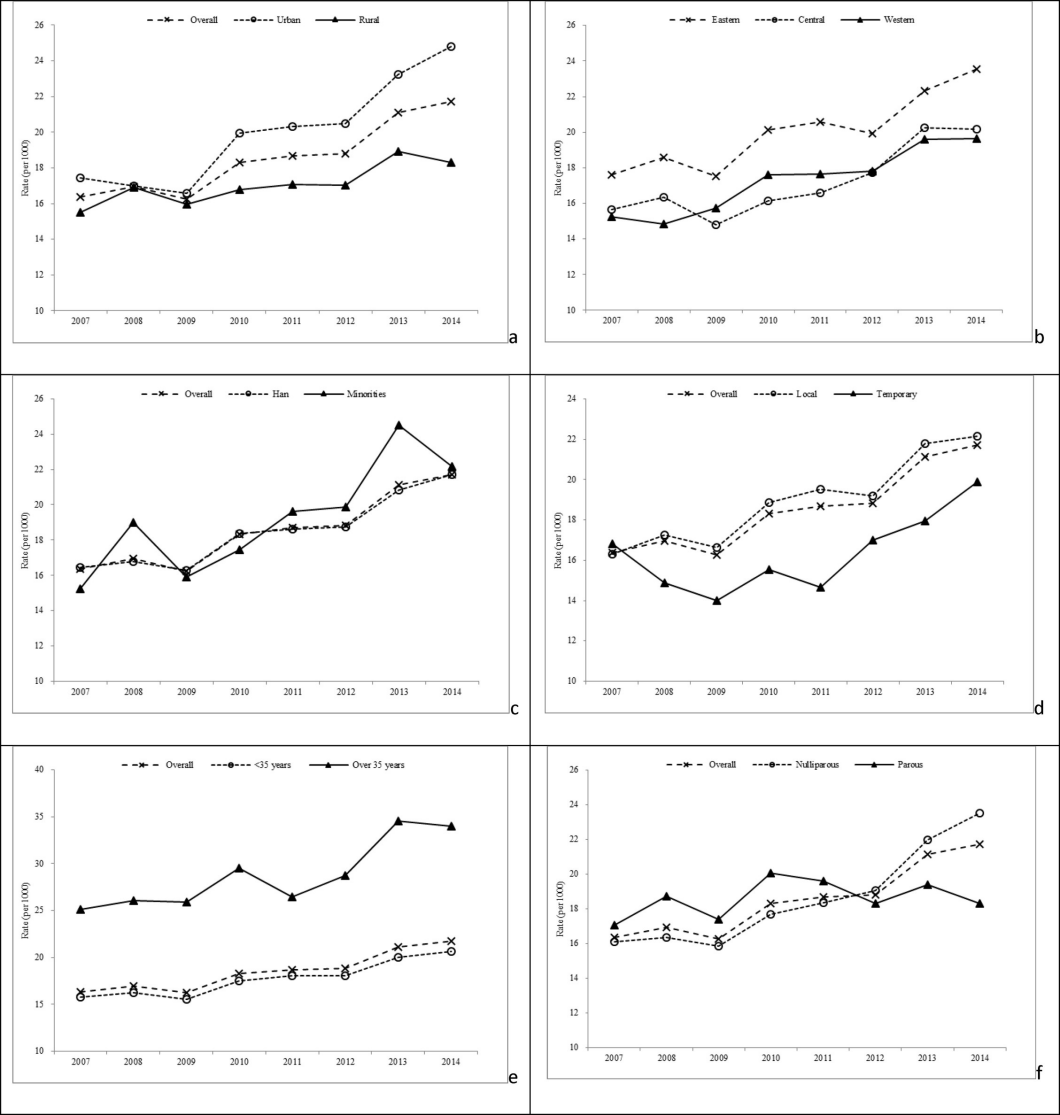
Time trends in twinning rates according to socio-demographic and infant characteristics in China, 2007–2014. Stratified by: a) urban-rural classification, b) geographic region, c) maternal ethnicity, d) maternal residence registration, e) maternal age, and f) parity.

Fig 1 and S2 Table show the time trends in twinning rates for specific stratification factors. The overall rate increased by 32.3% from 16.4‰ in 2007 to 21.7‰ in 2014. Greater increases were identified in urban vs rural groups (42.2% vs 18.2%), in eastern vs central and western regions (33.7% vs 28.8% and 29.0%), for local vs temporal household registrations (35.7% vs 18.3%), in minorities vs the Han nationality (45.7% vs 32.0%), in older vs younger maternal age groups (35.5% vs 30.9%), and in nulliparous vs parous women (46.3% vs 7.5%). Moreover, the estimated rates for dizygotic (9.9‰) and monozygotic (8.9‰) twins also varied significantly by birth area, geographic region, residence registration and maternal age (S3 Table). The temporal trends in dizygotic rates were highly similar to those observed in overall twinning rates (Fig 1, S1 Fig, S4 Table and S5 Table). Moreover, there was a sharper rise in dizygotic twinning rates in urban groups and women aged 35 years or older than in rural groups and younger women, respectively.

### Perinatal and neonatal mortality in twins

Table 2 presents stillbirth rate, perinatal mortality rate, early neonatal death rate, neonatal death rate in twins. The overall stillbirth rate and perinatal mortality rate were 14.1 and 26.1 per 1000 total births, while the rates for early neonatal death and neonatal death were 12.2 and 15.7 per 1000 live births, respectively. The perinatal mortality rate and neonatal death rate varied significantly by gestational age and birth weight, with higher rates in preterm and low-birth-weight twins. Twins from ethnic minority groups had a higher stillbirth rate and perinatal mortality rate than those from the Han nationality. Twins with birth defects were reported in 14.0% of perinatal deaths and 16.9% of neonatal deaths and had markedly higher perinatal mortality rate (131.6‰) and neonatal death rate (100.8‰). Compared with those in urban areas, in eastern or central regions and of male-female combination, twins in rural areas, in western region and of like-sexed combination had a higher stillbirth rate, perinatal mortality rate, early neonatal death rate and neonatal death rate.

**Table 2:** Perinatal and neonatal mortality of Chinese twins according to maternal and infant characteristics.

Group	Stillbirth		Perinatal mortality		Early neonatal death		Neonatal mortality	
	N (%)	Rate [‰,(95%CI)]	N (%)	Rate [‰,(95%CI)]	N (%)	Rate [‰,(95%CI)]	N (%)	Rate [‰,(95%CI)]
Birth area								
Urban	302(43.8)	11.5 (10.2,12.8)	562 (44.0)	21.3 (19.6,23.1)	260 (44.1)	10.0 (8.8,11.2)	334 (44.0)	12.8 (11.4,14.2)
Rural	387(56.2)	17.1 (15.4,18.8)	716 (56.0)	31.6 (29.3,33.9)	329 (55.9)	14.8 (13.2,16.4)	425 (56.0)	19.1 (17.3,20.9)
Geographic region								
Eastern	299(43.4)	12.0 (10.7,13.4)	570 (44.6)	23.0 (21.1,24.8)	271 (46.0)	11.0 (9.7,12.4)	347 (45.7)	14.1 (12.7,15.6)
Central	180(26.1)	13.9 (11.9,16.0)	325 (25.4)	25.2 (22.4,27.9)	145 (24.6)	11.4 (9.5,13.2)	189 (24.9)	14.8 (12.7,17.0)
Western	210(30.5)	18.6 (16.1,21.1)	383 (30.0)	34.0 (30.6,37.4)	173 (29.4)	15.6 (13.3,18)	223 (29.4)	20.1 (17.5,22.8)
Residence registration								
Local	606(88.0)	14.4 (13.2,15.5)	1116 (87.3)	26.5 (24.9,28.0)	510 (86.6)	12.3 (11.2,13.3)	655 (86.3)	15.8 (14.6,17.0)
Temporary	83(12.0)	12.1 (9.5,14.7)	162 (12.7)	23.6 (19.9,27.2)	79 (13.4)	11.6 (9.1,14.2)	104 (13.7)	15.3 (12.4,18.3)
Gender*								
Male	363(52.7)	14.3 (12.8,15.8)	661 (51.7)	26.1 (24.1,28.0)	298 (50.6)	11.9 (10.6,13.3)	388 (51.1)	16.5 (14.9,18.1)
Female	318(46.2)	13.5 (12.0,14.9)	606 (47.4)	25.6 (23.6,27.7)	288 (48.9)	12.3 (10.9,13.8)	368 (48.5)	16.5 (14.9,18.2)
Sex combination								
Male-female	97(14.1)	7.5 (6.0,9.0)	224 (17.5)	17.3 (15.1,19.6)	127 (21.6)	9.9 (8.2,11.6)	165 (21.7)	12.9 (10.9,14.8)
Male-male	318(46.2)	16.8 (15.0,18.7)	555 (43.4)	29.3 (26.9,31.8)	237 (40.2)	12.7 (11.1,14.4)	306 (40.3)	16.5 (14.6,18.3)
Female-female	274(39.8)	15.9 (14.0,17.8)	499 (39)	29.0 (26.5,31.6)	225 (38.2)	13.3 (11.6,15.0)	288 (37.9)	17.0 (15.1,19.0)
Ethnicity								
Han	617(89.6)	13.6 (12.5,14.6)	1159 (90.7)	25.5 (24.0,27.0)	542 (92.0)	12.1 (11.1,13.1)	694 (91.4)	15.5 (14.3,16.6)
Minorities	72(10.4)	20.2 (15.6,24.9)	119 (9.3)	33.4 (27.4,39.4)	47 (8.0)	13.5 (9.6,17.3)	65 (8.6)	18.6 (14.1,23.2)
Maternal age (yrs)^$^								
<35	621(90.1)	14.3 (13.2,15.4)	1150 (90.0)	26.5 (24.9,28.0)	529 (89.8)	12.4 (11.3,13.4)	675 (88.9)	15.8 (14.6,17.0)
≥35	68(9.9)	12.3 (9.4,15.2)	127 (9.9)	22.9 (19.0,26.9)	59 (10.0)	10.8 (8.0,13.5)	82 (10.8)	15.0 (11.8,18.2)
Parity								
nulliparous	527(76.5)	15.1 (13.8,16.4)	947 (74.1)	27.1 (25.3,28.8)	420 (71.3)	12.2 (11.0,13.4)	533 (70.2)	15.5 (14.2,16.8)
parous	162(23.5)	11.5 (9.8,13.3)	331 (25.9)	23.6 (21.1,26.1)	169 (28.7)	12.2 (10.4,14.0)	226 (29.8)	16.3 (14.2,18.4)
Birth defects								
No	599(86.9)	12.6 (11.6,13.6)	1099 (86.0)	23.1 (21.7,24.4)	500 (84.9)	10.6 (9.7,11.6)	631 (83.1)	13.4 (12.4,14.5)
Yes	90(13.1)	66.2 (52.5,79.8)	179 (14.0)	131.6 (112.3,150.9)	89 (15.1)	70.1 (55.5,84.6)	128 (16.9)	100.8 (83.3,118.2)
Birth weight (g) ^#^								
<1500g	243(35.3)	154.1 (134.7,173.5)	520 (40.7)	329.7 (301.4,358.1)	277 (47.0)	207.6 (183.2,232.1)	337 (44.4)	252.6 (225.7,279.6)
1500-2499g	314(45.6)	15.8 (14.1,17.6)	563 (44.1)	28.4 (26.0,30.7)	249 (42.3)	12.7 (11.2,14.3)	329 (43.3)	16.8 (15.0,18.7)
≥2500g	106(15.4)	3.9 (3.1,4.6)	162 (12.7)	5.9 (5.0,6.8)	56 (9.5)	2.0 (1.5,2.6)	85 (11.2)	3.1 (2.4,3.8)
Overall low birth weight	557(80.8)	26.0 (23.8,28.2)	1083 (84.7)	50.5 (47.5,53.5)	526 (89.3)	25.2 (23,27.4)	666 (87.7)	31.9 (29.5,34.3)
Gestation age (weeks) ^&^								
<32	179(26.0)	114.7 (97.9,131.6)	481 (37.6)	308.3 (280.8,335.9)	302 (51.3)	218.7 (194.0,243.3)	364 (48.0)	263.6 (236.5,290.7)
32–36	295(42.8)	18.4 (16.3,20.5)	503 (39.4)	31.4 (28.6,34.1)	208 (35.3)	13.2 (11.4,15.0)	277 (36.5)	17.6 (15.5,19.7)
≥37	215(31.2)	6.9 (5.9,7.8)	294 (23.0)	9.4 (8.3,10.4)	79 (13.4)	2.5 (2.0,3.1)	117 (15.4)	3.8 (3.1,4.4)
Overall preterm births	474(68.8)	27.0 (24.5,29.4)	984 (77.0)	56.0 (52.5,59.5)	510 (86.6)	29.8 (27.2,32.4)	641 (84.5)	37.5 (34.6,40.4)
Total	689(100.0)	14.1 (13.0,15.1)	1278 (100.0)	26.1 (24.6,27.5)	589 (100.0)	12.2 (11.2,13.2)	759 (100.0)	15.7 (14.6,16.8)

*18 infants with unspecified sex

^$^56 with unspecified maternal age

^&^126 with unknown birth weight, and ^&^77 with unspecified gestational age were excluded from the analysis.

The confidence interval of rate was calculated based on Poisson distribution.

**Fig 2:**
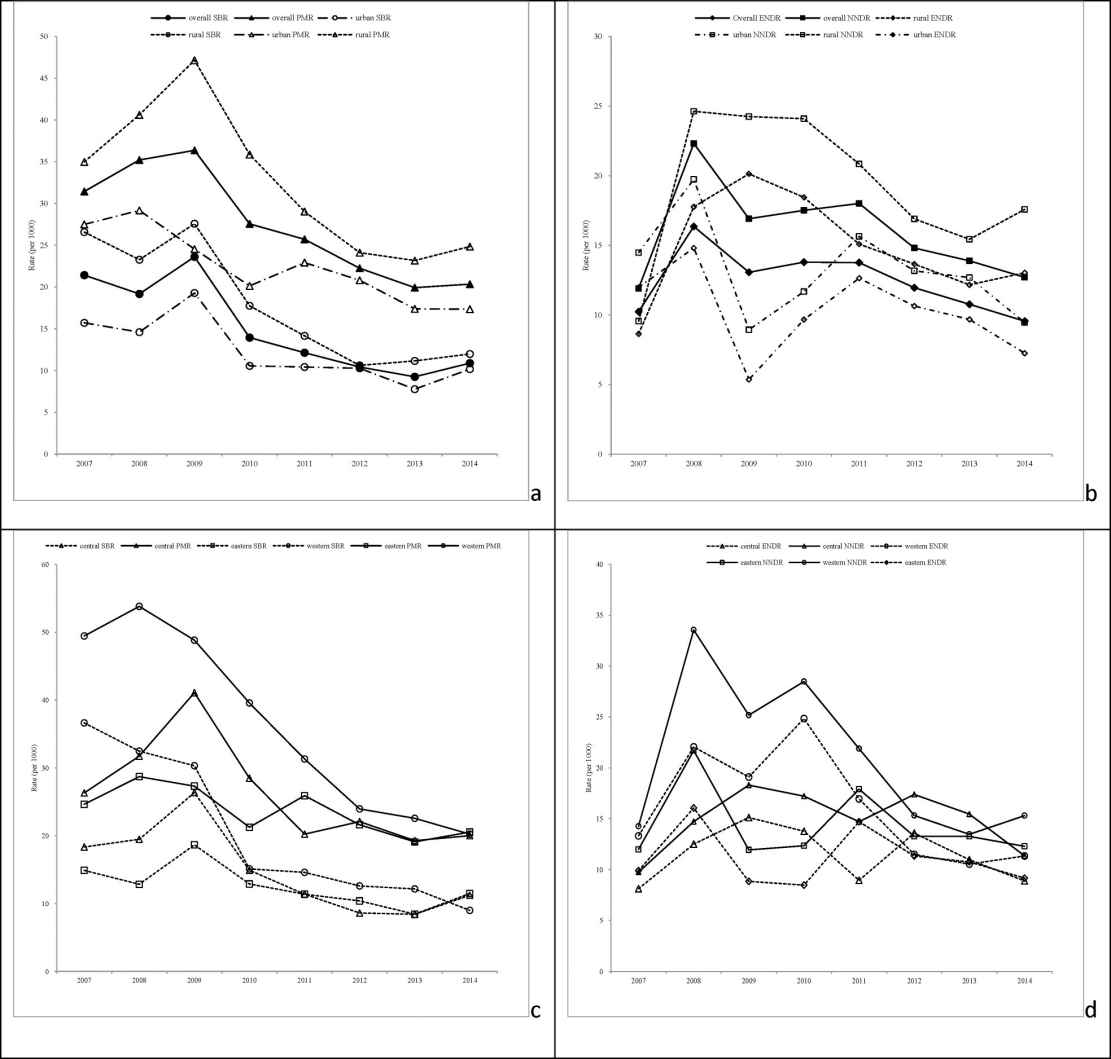
Time trends in stillbirth rate and the perinatal, early neonatal and neonatal mortality rates in Chinese twins. SBR, PMR, ENDR and NNDR represent stillbirth rate, perinatal mortality rate, early neonatal death rate and neonatal death rate, respectively. a. SBR and PMR by birth area; b. ENDR and NNDR by birth area; c. SBR and PMR by geographic region; d. ENDR and NNDR by geographic region.

In terms of time trends in mortality, the stillbirth rate decreased by 49.2% from 21.4‰ in 2007 to 10.9‰ in 2014, and the perinatal mortality rate declined by 35.3% from 31.4‰ to 20.3‰ during the same period. Urban-rural and geographic differences were still prominent in the time trends of these two indicators. Greater decreases of stillbirth rate existed in rural areas (54.9%) than in urban areas (35.3%), and in western regions (75.5%) than in central regions (38.8%) and eastern regions (23.9%). For perinatal mortality rate, larger decreases were found in urban areas (36.9%) than in rural areas (29.0%) and in western regions (59.1%) than in central regions (22.9%) and eastern regions (16.6%). The early neonatal death rate and neonatal death rate among twins also showed a downward trend. However, the downward trend in the neonatal death rate was evident only in urban areas when analyzing by birth area, while the downward trends in the early neonatal death rate and neonatal death rate were significant only in western regions when analyzing by region (Fig 2 and S6 Table).

## Discussion

This study found that the twinning rate in Chinese population was 18.8 per 1000 births and presented an upward trend over 2007–2014. The stillbirth rate, perinatal mortality rate, early neonatal death rate and neonatal death rate in twins declined substantially during this period but remained at a high level (i.e., 26.1‰ for the perinatal mortality rate and 15.7‰ for the neonatal death rate). Moreover, significant urban-rural and regional disparities persisted in the birth rates and mortality rates of twins, as well as in the time trends of these indicators.

We found that the overall twinning rate of 18.8‰ was similar to previous rates observed in China (from 13 to 20.5 per 1000 births) [22–24] and other Asian countries (i.e. 19.3‰ in 2002 in Korea [25] and 19.5‰ in 2009 in Japan [26]) but lower than those in some western countries (i.e., 27.3‰ in England and Wales, 28.3‰ in France in 1997 [6], and 33.3‰ in 2009 in the United States [1]). Consistent with the literature, twinning rates varied by maternal age, gender, and ethnicity [27–29]. Genetic background may contribute to the female predominance and ethnic variation observed in twinning rates, while environmental determinants may explain the differences observed between groups by birth area, region, maternal age and residence [22, 24].

Temporal trends in twinning rates have not been well documented in China [12, 24]. The rising twinning rates in developed countries have been primarily attributed to the increasing percentage of advanced maternal age and infertility treatments such as assisted reproductive technologies [1, 25, 30–35]. Approximately 1/4 to 1/3 of the increase in multiple births can be attributed to advanced maternal age, while 2% to 24% of twins may be due to the use of assisted reproductive technologies [5]. Similarly, the upward trend in the twinning rate observed in our study could be also related to advanced maternal age and the use of assisted reproductive technologies, with advanced maternal age alone potentially explaining 15.7% of the increase in all twins. The urban-rural and regional differences observed in the overall twinning and dizygotic rates provide additional supporting evidence for this possibility (Table 1 & S3 Table). In China, women in urban areas or the eastern region usually have higher socioeconomic statuses as well as better awareness and access to prenatal care than those in rural areas or the western regions [18, 20, 22]. According to a recent study, approximately 1% of all births resulted from assisted reproductive technologies in mainland China in 2011 and 17.6% of them were multiple births. Nearly one-third of women who use assisted reproductive technologies are over 35 years of age, and the majority live in urban areas or in the eastern region [36]. Notably, there was a sharp increase in twinning rates from 2009 to 2014, and this increase is more evident in urban areas, in the eastern region, and in the advanced maternal age group. During this same period, the percentage of infants born to parous women rose from 26.1% in 2009 to 30.3% in 2012 and to 34.2% in 2014. Collectively, these phenomena might be related to changes in China’s one-child policy, which began to loosen in 2009 and was officially ended in 2014. Therefore, the observed changes in the twinning rate are likely influenced by many social and health-related factors.

Prior to this study, there have been no reliable national estimates of the perinatal mortality rate and neonatal death rate among twins in China. The perinatal mortality rates in our sample were higher than those reported in developed countries (i.e., 6.6‰ in the Netherlands [37], 21.5‰ in Ireland [38] and 20.1‰ in the United States) and lower than those reported in most low- or middle-income countries [11]. Indeed, the observed rate might be even higher if the criterion of perinatal deaths in some countries were adopted (fetal deaths prior to gestational week 28) [9, 39]. The early neonatal death rate (12.2‰) and neonatal death rate (15.7‰) were also markedly greater than those in some western and Asian populations [40]. Overall there was a downward trend in the overall perinatal and neonatal mortalities of twins from China. Urban-rural and regional disparities were found in perinatal mortality rate, neonatal death rate as well as in their temporal trends. Substantial declines in the perinatal mortality rate in rural area and the western region may be attributed to effective intervention programs in poverty-stricken areas launched by China central government [41, 42]. However, large gaps in perinatal and neonatal mortality of twins between Chinese and other populations suggest an urgent need to improve perinatal and neonatal health care.

## Conclusions

To the best of our knowledge, this is the first study to generate a nationally representative population-based epidemiological report on the birth rates and clinical consequences of twins in China. The major strength of the current study is the quality of data that were abstracted from a large and well-documented national registry with good representation of China’s demographics and geographic regions. Furthermore, the ethnic, urban-rural and geographic distributions are proportional to those observed in the 2010 National Census (http://www.stats.gov.cn/tjgb/rkpcgb/). Birth outcomes and other key variables were obtained from medical records by trained surveillance staff, which minimized misclassification of clinical outcomes.

One limitation of this study is the lack of data on some obstetric and fetal characteristics, including information on zygote, chorionicity, amnionicity, fetal presentation, mode of delivery, pregnancy complications, etc. However, the socio-demographic variables included in our analysis can well reflect disparities across a range of maternal and child health indicators, which have been validated in previous studies in China [16, 18, 19]. Another limitation is that we were unable to analyze the underlying cause of death in twins due to limited data, but this was not a major focus of this study. Underreporting could have also influenced the accuracy of our data registry, though the effects were likely minimal due to strict data quality control procedures adopted by the NPBDSS.

In summary, twins represented 1.9% of all births in our study population and made an increasing contribution to total early neonatal deaths and neonatal deaths in China. Temporal changes in adverse perinatal outcomes of twins are generally consistent with those observed in developed countries that are experiencing increased twinning rates, and these changes display large urban-rural and geographic disparities. Twinning rates in China continue to rise resulting in a major challenge for policy makers who seek to reduce infant and child mortality rates through improvements in equity in healthcare. Our study provides a national perspective on twinning rates and perinatal outcomes which have, to date, never been systematically analyzed. These findings will be of great value for future studies on demographic transitions and birth outcomes in China and serve as a valuable reference for other developing countries.

## Supporting information

S1 Table Maternal and infant characteristics associated with twinning rates. (DOCX)

S2 Table Time trends in twinning rates in China, 2007–2014. (DOCX)

S3 Table Twinning rates by zygotic type in Chinese population, 2007–2014. (DOCX)

S4 Table Time trends in dizygotic twinning rates in China, 2007–2014. (DOCX)

S5 Table Time trends in monozygotic twinning rates in China, 2007–2014. (DOCX)

S6 Table Time trends in stillbirth rate and the perinatal, early neonatal and neonatal mortality rates in Chinese twins. (DOCX)

S1 Fig Time trends in dizygotic and monozygotic twinning rates by selected maternal and infant factors in China, 2007–2014. Stratified by: a) urban-rural classification, b) geographic region, c) maternal residence registration, d) maternal ethnicity, e) maternal age, and f) parity.
(TIFF)
