# Supplementary material for: Temporal trends in the birth rates and perinatal mortality of twins: A population-based study in China
Source: PLoS One. 2019 Jan 16;14(1):e0209962. doi: 10.1371/journal.pone.0209962 (PMC6334899; doi:10.1371/journal.pone.0209962)
Supplement: S1 Table — (DOCX) [file pone.0209962.s001.docx]

**S1 Table Maternal and infant characteristics associated with twinning rates**

| Variables | Variable assignment | Coefficient (95%CI) | S.E. | Z | *P* |
| --- | --- | --- | --- | --- | --- |
| Birth area | urban=1, rural^*^=2 | 0.195(0.174,0.215) | 0.010 | 18.76 | 0.000 |
| Geographic region | eastern=1, | 0.152(0.127,0.176) | 0.012 | 12.20 | 0.000 |
|  | central=2, western^*^=3 | 0.031(0.005,0.058) | 0.014 | 2.30 | 0.021 |
| Residence registration | local^*^=1, temporal=2 | -0.277(-0.305,-0.250) | 0.014 | -19.54 | 0.000 |
| Gender | male=1, female^*^=2 | -0.060(-0.078,-0.043) | 0.009 | -6.74 | 0.000 |
| Ethnicity | Han^*^=1, minority=2 | 0.133(0.097,0.170) | 0.019 | 7.17 | 0.000 |
| Maternal age (yrs) | <35^*^=1, ≥35=2 | 0.513(0.482,0.543) | 0.015 | 33.38 | 0.000 |
| Parity | nulliparous^*^=1, parous=2 | -0.022(-0.044,0.001) | 0.011 | -1.89 | 0.058 |

^*^ these were set as reference. Counted by poisson regression.
