# Supplementary material for: Temporal trends in the birth rates and perinatal mortality of twins: A population-based study in China
Source: PLoS One. 2019 Jan 16;14(1):e0209962. doi: 10.1371/journal.pone.0209962 (PMC6334899; doi:10.1371/journal.pone.0209962)
Supplement: S2 Table — (DOCX) [file pone.0209962.s002.docx]

**S2 Table Time trends in twinning rates in China, 2007-2014.**

| Group | 2007 | | 2008 | | 2009 | | 2010 | | 2011 | | 2012 | | 2013 | | 2014 | |
| --- | --- | --- | --- | --- | --- | --- | --- | --- | --- | --- | --- | --- | --- | --- | --- | --- |
|  | No. | Rate(‰) | No. | Rate(‰) | No. | Rate(‰) | No. | Rate(‰) | No. | Rate(‰) | No. | Rate(‰) | No. | Rate(‰) | No. | Rate(‰) |
| Birth area |  |  |  |  |  |  |  |  |  |  |  |  |  |  |  |  |
| urban | 2036 | 17.4 | 2262 | 17.0 | 2282 | 16.6 | 3030 | 20.0 | 3359 | 20.3 | 3989 | 20.5 | 4372 | 23.2 | 5016 | 24.8 |
| rural | 2258 | 15.5 | 2536 | 16.9 | 2502 | 16.0 | 2704 | 16.8 | 2824 | 17.1 | 3110 | 17.0 | 3408 | 18.9 | 3340 | 18.3 |
| Geographic region |  |  |  |  |  |  |  |  |  |  |  |  |  |  |  |  |
| eastern | 1948 | 17.6 | 2334 | 18.6 | 2306 | 17.5 | 2870 | 20.1 | 3164 | 20.6 | 3656 | 19.9 | 4030 | 22.3 | 4528 | 23.5 |
| central | 1254 | 15.7 | 1386 | 16.3 | 1290 | 14.8 | 1474 | 16.1 | 1582 | 16.6 | 1856 | 17.7 | 2022 | 20.2 | 2048 | 20.2 |
| western | 1092 | 15.2 | 1078 | 14.8 | 1188 | 15.7 | 1390 | 17.6 | 1437 | 17.7 | 1587 | 17.8 | 1728 | 19.6 | 1780 | 19.7 |
| Residence registration | |  |  |  |  |  |  |  |  |  |  |  |  |  |  |  |
| local | 3862 | 16.3 | 4226 | 17.3 | 4192 | 16.6 | 4968 | 18.8 | 5370 | 19.5 | 5968 | 19.2 | 6596 | 21.8 | 6966 | 22.1 |
| temporal | 428 | 16.8 | 572 | 14.9 | 592 | 14.0 | 766 | 15.5 | 813 | 14.7 | 1131 | 17.0 | 1184 | 18.0 | 1390 | 19.9 |
| Ethnicity |  |  |  |  |  |  |  |  |  |  |  |  |  |  |  |  |
| Han | 4047 | 16.4 | 4443 | 16.8 | 4457 | 16.3 | 5343 | 18.4 | 5734 | 18.6 | 6575 | 18.7 | 7123 | 20.8 | 7746 | 21.7 |
| minority | 247 | 15.2 | 355 | 19.0 | 327 | 15.9 | 391 | 17.4 | 449 | 19.6 | 524 | 19.9 | 657 | 24.5 | 610 | 22.2 |
| Maternal age (yrs) |  |  |  |  |  |  |  |  |  |  |  |  |  |  |  |  |
| <35 | 3860 | 15.8 | 4296 | 16.3 | 4246 | 15.5 | 5078 | 17.5 | 5545 | 18.1 | 6305 | 18.0 | 6794 | 20.0 | 7314 | 20.7 |
| ≥35 | 396 | 25.1 | 502 | 26.1 | 536 | 25.9 | 656 | 29.5 | 638 | 26.5 | 788 | 28.8 | 980 | 34.6 | 1038 | 34.0 |
| Parity |  |  |  |  |  |  |  |  |  |  |  |  |  |  |  |  |
| nulliparous | 3240 | 16.1 | 3480 | 16.4 | 3448 | 15.9 | 4050 | 17.7 | 4339 | 18.3 | 5009 | 19.0 | 5468 | 22.0 | 5946 | 23.5 |
| parous | 1038 | 17.0 | 1318 | 18.7 | 1334 | 17.4 | 1684 | 20.1 | 1844 | 19.6 | 2090 | 18.3 | 2312 | 19.4 | 2408 | 18.3 |
