# Supplementary material for: Temporal trends in the birth rates and perinatal mortality of twins: A population-based study in China
Source: PLoS One. 2019 Jan 16;14(1):e0209962. doi: 10.1371/journal.pone.0209962 (PMC6334899; doi:10.1371/journal.pone.0209962)
Supplement: S3 Table — (DOCX) [file pone.0209962.s003.docx]

**S3 Table Twin rates by zygotic type in Chinese population, 2007-2014.**

| Group | N (%) | |  | Rate[‰,(95%CI)] | |
| --- | --- | --- | --- | --- | --- |
|  | MZ | DZ |  | MZ | DZ |
| Birth area |  |  |  |  |  |
| urban | 12454 (47.3) | 13892 (52.7) |  | 9.7(9.5,9.8) | 10.8(10.6,11.0) |
| rural | 10746 (47.4) | 11936 (52.6) |  | 8.1(8.0,8.3) | 9.0(8.9,9.2) |
| Geographic region |  |  |  |  |  |
| eastern | 11340 (45.7) | 13496 (54.3) |  | 9.3 (9.1, 9.5) | 11.1 (10.9, 11.2) |
| central | 6320 (48.9) | 6592 (51.1) |  | 8.5(8.3, 8.7) | 8.8 (8.6, 9.1) |
| western | 5540 (49.1) | 5740 (50.9) |  | 8.5(8.3, 8.8) | 8.9 (8.6, 9.1) |
| Residence registration^#^ |  |  |  |  |  |
| local | 19282 (45.7) | 22866 (54.3) |  | 9.5 (9.2, 9.8 ) | 7.2 (6.9, 7.4 ) |
| temporal | 3914 (56.9) | 2962 (43.1) |  | 8.8 (8.6, 8.9 ) | 10.4 ( 10.3 , 10.5 ) |
| Gender^*^ |  |  |  |  |  |
| male | 12455 (49.1) | 12914 (50.9) |  | 8.9 (8.8, 9.1) | 9.3 (9.1, 9.4) |
| female | 10739 (45.4) | 12902 (54.6) |  | 8.8 (8.6, 9.0) | 10.6 (10.4, 10.8) |
| Ethnicity^ǂ^ |  |  |  |  |  |
| Han | 21512 (47.3) | 23956 (52.7) |  | 8.8 (8.7, 9.0) | 9.8 (9.7, 10.0) |
| minority | 1688 (47.4) | 1872 (52.6) |  | 9.3 (8.9, 9.7) | 10.3 (9.9, 10.8) |
| Maternal age (yrs)^$^ |  |  |  |  |  |
| <35 | 21214 (48.8) | 22224 (51.2) |  | 8.8 (8.6, 8.9) | 9.2 (9.1, 9.3) |
| ≥35 | 1962 (35.5) | 3572 (64.5) |  | 10.4 (10.0, 10.9) | 19.0 (18.4, 19.6) |
| Parity^&^ |  |  |  |  |  |
| nulliparous | 16300 (46.6) | 18680 (53.4) |  | 9.2 (9.0, 9.4 ) | 9.5 (9.3, 9.7 ) |
| parous | 6888 (49.1) | 7140 (50.9) |  | 8.8 ( 8.6, 8.9 ) | 10.0 (9.9, 10.2 ) |
| Total | 23192 (47.3) | 25836 (52.7) |  | 8.9 (8.8, 9.0) | 9.9 (9.8,10.0) |

MZ and DZ represent monozygotic and dizygotic twins. MZ and DZ twin pairs was estimated by using the Weinberg’s differential method, then multiplied by 2 to get the number of twin individuals. The confidence interval of rate was calculated based on Poisson distribution.

^#^292 infants with unspecified maternal residence, ^*^675 births with unknown or unspecified gender, ^ǂ^7 births with unknown maternal ethnicity, ^$^3166 births with unknown maternal age, and ^&^955 births with unknown parity were excluded.

Both MZ and DZ rate varied significantly by birth area, geographic region, residence registration and maternal age (all p value＜0.001). Moreover, MZ rate differed by maternal ethnicity (p＜0.05).
