# Supplementary material for: Temporal trends in the birth rates and perinatal mortality of twins: A population-based study in China
Source: PLoS One. 2019 Jan 16;14(1):e0209962. doi: 10.1371/journal.pone.0209962 (PMC6334899; doi:10.1371/journal.pone.0209962)
Supplement: S4 Table — (DOCX) [file pone.0209962.s004.docx]

**S4 Table. Time trends in dizygotic twinning rates in China, 2007-2014.**

| Group | 2007 | | 2008 | | 2009 | | 2010 | | 2011 | | 2012 | | 2013 | | 2014 | |
| --- | --- | --- | --- | --- | --- | --- | --- | --- | --- | --- | --- | --- | --- | --- | --- | --- |
|  | No. | Rate(‰) | No. | Rate(‰) | No. | Rate(‰) | No. | Rate(‰) | No. | Rate(‰) | No. | Rate(‰) | No. | Rate(‰) | No. | Rate(‰) |
| Birth area |  |  |  |  |  |  |  |  |  |  |  |  |  |  |  |  |
| urban | 848 | 7.3 | 1080 | 8.1 | 1084 | 7.9 | 1556 | 10.2 | 1830 | 11.1 | 2118 | 10.9 | 2628 | 14.0 | 2748 | 13.6 |
| rural | 1120 | 7.7 | 1452 | 9.7 | 1292 | 8.2 | 1420 | 8.8 | 1436 | 8.7 | 1600 | 8.8 | 1940 | 10.8 | 1676 | 9.2 |
| Geographic region |  |  |  |  |  |  |  |  |  |  |  |  |  |  |  |  |
| eastern | 916 | 8.3 | 1280 | 10.2 | 1192 | 9.1 | 1584 | 11.1 | 1756 | 11.4 | 1880 | 10.2 | 2428 | 13.5 | 2460 | 12.8 |
| central | 548 | 6.8 | 648 | 7.6 | 584 | 6.7 | 748 | 8.2 | 808 | 8.5 | 960 | 9.2 | 1164 | 11.7 | 1132 | 11.2 |
| western | 504 | 7.0 | 604 | 8.3 | 600 | 7.9 | 644 | 8.2 | 702 | 8.6 | 878 | 9.9 | 976 | 11.1 | 832 | 9.2 |
| Residence registration | |  |  |  |  |  |  |  |  |  |  |  |  |  |  |  |
| local | 1838 | 7.8 | 2334 | 9.5 | 2154 | 8.5 | 2682 | 10.2 | 2914 | 10.6 | 3232 | 10.4 | 3986 | 13.2 | 3726 | 11.8 |
| temporal | 130 | 5.1 | 198 | 5.2 | 222 | 5.2 | 294 | 6.0 | 352 | 6.4 | 486 | 7.3 | 582 | 8.8 | 698 | 10.0 |
| Ethnicity |  |  |  |  |  |  |  |  |  |  |  |  |  |  |  |  |
| Han | 1852 | 7.5 | 2360 | 8.9 | 2232 | 8.2 | 2790 | 9.6 | 3046 | 9.9 | 3404 | 9.7 | 4148 | 12.1 | 4124 | 11.6 |
| minority | 116 | 7.1 | 172 | 9.2 | 144 | 7.0 | 186 | 8.3 | 220 | 9.6 | 314 | 11.9 | 420 | 15.7 | 300 | 10.9 |
| Maternal age (yrs) |  |  |  |  |  |  |  |  |  |  |  |  |  |  |  |  |
| <35 | 1744 | 7.1 | 2208 | 8.4 | 2072 | 7.6 | 2568 | 8.8 | 2830 | 9.2 | 3182 | 9.1 | 3912 | 11.5 | 3708 | 10.5 |
| ≥35 | 200 | 12.7 | 324 | 16.8 | 304 | 14.7 | 408 | 18.4 | 436 | 18.1 | 536 | 19.6 | 652 | 23.0 | 712 | 23.3 |
| Parity |  |  |  |  |  |  |  |  |  |  |  |  |  |  |  |  |
| nulliparous | 1444 | 7.2 | 1732 | 8.1 | 1684 | 7.7 | 2124 | 9.3 | 2346 | 9.9 | 2666 | 10.1 | 3388 | 13.6 | 3296 | 13.0 |
| parous | 516 | 8.5 | 800 | 11.4 | 692 | 9.0 | 852 | 10.1 | 920 | 9.8 | 1052 | 9.2 | 1180 | 9.9 | 1128 | 8.6 |
