# Supplementary material for: Temporal trends in the birth rates and perinatal mortality of twins: A population-based study in China
Source: PLoS One. 2019 Jan 16;14(1):e0209962. doi: 10.1371/journal.pone.0209962 (PMC6334899; doi:10.1371/journal.pone.0209962)
Supplement: S5 Table — (DOCX) [file pone.0209962.s005.docx]

**S5 Table Time trends in monozygotic twinning rates in China, 2007-2014.**

| Group | 2007 | | 2008 | | 2009 | | 2010 | | 2011 | | 2012 | | 2013 | | 2014 | |
| --- | --- | --- | --- | --- | --- | --- | --- | --- | --- | --- | --- | --- | --- | --- | --- | --- |
|  | No. | Rate(‰) | No. | Rate(‰) | No. | Rate(‰) | No. | Rate(‰) | No. | Rate(‰) | No. | Rate(‰) | No. | Rate(‰) | No. | Rate(‰) |
| Birth area |  |  |  |  |  |  |  |  |  |  |  |  |  |  |  |  |
| urban | 1188 | 10.2 | 1182 | 8.9 | 1198 | 8.7 | 1474 | 9.7 | 1529 | 9.3 | 1871 | 9.6 | 1744 | 9.3 | 2268 | 11.2 |
| rural | 1138 | 7.8 | 1084 | 7.2 | 1210 | 7.7 | 1284 | 8.0 | 1388 | 8.4 | 1510 | 8.3 | 1468 | 8.1 | 1664 | 9.1 |
| Geographic region |  |  |  |  |  |  |  |  |  |  |  |  |  |  |  |  |
| eastern | 1032 | 9.3 | 1054 | 8.4 | 1114 | 8.5 | 1286 | 9.0 | 1408 | 9.1 | 1776 | 9.7 | 1602 | 8.9 | 2068 | 10.7 |
| central | 706 | 8.8 | 738 | 8.7 | 706 | 8.1 | 726 | 7.9 | 774 | 8.1 | 896 | 8.6 | 858 | 8.6 | 916 | 9.0 |
| western | 750 | 10.5 | 782 | 10.8 | 690 | 9.1 | 830 | 10.5 | 880 | 10.8 | 978 | 11.0 | 1046 | 11.9 | 1216 | 13.4 |
| Residence registration | |  |  |  |  |  |  |  |  |  |  |  |  |  |  |  |
| local | 2024 | 8.6 | 1892 | 7.7 | 2038 | 8.1 | 2286 | 8.7 | 2456 | 8.9 | 2736 | 8.8 | 2610 | 8.6 | 3240 | 10.3 |
| temporal | 298 | 11.7 | 374 | 9.7 | 370 | 8.7 | 472 | 9.6 | 461 | 8.3 | 645 | 9.7 | 602 | 9.1 | 692 | 9.9 |
| Ethnicity |  |  |  |  |  |  |  |  |  |  |  |  |  |  |  |  |
| Han | 2195 | 8.9 | 2083 | 7.9 | 2225 | 8.1 | 2553 | 8.8 | 2688 | 8.7 | 3171 | 9.0 | 2975 | 8.7 | 3622 | 10.1 |
| minority | 131 | 8.1 | 183 | 9.8 | 183 | 8.9 | 205 | 9.1 | 229 | 10.0 | 210 | 8.0 | 237 | 8.8 | 310 | 11.3 |
| Maternal age (yrs) |  |  |  |  |  |  |  |  |  |  |  |  |  |  |  |  |
| <35 | 2116 | 8.7 | 2088 | 7.9 | 2174 | 8.0 | 2510 | 8.6 | 2715 | 8.9 | 3123 | 8.9 | 2882 | 8.5 | 3606 | 10.2 |
| ≥35 | 196 | 12.4 | 178 | 9.3 | 232 | 11.2 | 248 | 11.2 | 202 | 8.4 | 252 | 9.2 | 328 | 11.6 | 326 | 10.7 |
| Parity |  |  |  |  |  |  |  |  |  |  |  |  |  |  |  |  |
| nulliparous | 1796 | 8.9 | 1748 | 8.2 | 1764 | 8.1 | 1926 | 8.4 | 1993 | 8.4 | 2343 | 8.9 | 2080 | 8.4 | 2650 | 10.5 |
| parous | 522 | 8.6 | 518 | 7.4 | 642 | 8.4 | 832 | 9.9 | 924 | 9.8 | 1038 | 9.1 | 1132 | 9.5 | 1280 | 9.7 |
