# Supplementary material for: Temporal trends in the birth rates and perinatal mortality of twins: A population-based study in China
Source: PLoS One. 2019 Jan 16;14(1):e0209962. doi: 10.1371/journal.pone.0209962 (PMC6334899; doi:10.1371/journal.pone.0209962)
Supplement: S6 Table — (DOCX) [file pone.0209962.s006.docx]

**S6 Table Time trends in stillbirth rate and the perinatal, early neonatal and neonatal mortality rates in Chinese twins.**

| Group | urban | | rural | | eastern | | central | | western | |
| --- | --- | --- | --- | --- | --- | --- | --- | --- | --- | --- |
|  | No. | Rate(‰) | No. | Rate(‰) | No. | Rate(‰) | No. | Rate(‰) | No. | Rate(‰) |
| Stillbirth |  |  |  |  |  |  |  |  |  |  |
| 2007 | 32 | 15.7 | 60 | 26.6 | 29 | 14.9 | 23 | 18.3 | 40 | 36.6 |
| 2008 | 33 | 14.6 | 59 | 23.3 | 30 | 12.9 | 27 | 19.5 | 35 | 32.5 |
| 2009 | 44 | 19.3 | 69 | 27.6 | 43 | 18.6 | 34 | 26.4 | 36 | 30.3 |
| 2010 | 32 | 10.6 | 48 | 17.8 | 37 | 12.9 | 22 | 14.9 | 21 | 15.1 |
| 2011 | 35 | 10.4 | 40 | 14.2 | 36 | 11.4 | 18 | 11.4 | 21 | 14.6 |
| 2012 | 41 | 10.3 | 33 | 10.6 | 38 | 10.4 | 16 | 8.6 | 20 | 12.6 |
| 2013 | 34 | 7.8 | 38 | 11.2 | 34 | 8.4 | 17 | 8.4 | 21 | 12.2 |
| 2014 | 51 | 10.2 | 40 | 12.0 | 52 | 11.5 | 23 | 11.2 | 16 | 9.0 |
| Perinatal mortality |  |  |  |  |  |  |  |  |  |  |
| 2007 | 56 | 27.5 | 79 | 35.0 | 48 | 24.6 | 33 | 26.3 | 54 | 49.5 |
| 2008 | 66 | 29.2 | 103 | 40.6 | 67 | 28.7 | 44 | 31.7 | 58 | 53.8 |
| 2009 | 56 | 24.5 | 118 | 47.2 | 63 | 27.3 | 53 | 41.1 | 58 | 48.8 |
| 2010 | 61 | 20.1 | 97 | 35.9 | 61 | 21.3 | 42 | 28.5 | 55 | 39.6 |
| 2011 | 77 | 22.9 | 82 | 29.0 | 82 | 25.9 | 32 | 20.2 | 45 | 31.3 |
| 2012 | 83 | 20.8 | 75 | 24.1 | 79 | 21.6 | 41 | 22.1 | 38 | 23.9 |
| 2013 | 76 | 17.4 | 79 | 23.2 | 77 | 19.1 | 39 | 19.3 | 39 | 22.6 |
| 2014 | 87 | 17.3 | 83 | 24.9 | 93 | 20.5 | 41 | 20.0 | 36 | 20.2 |
| Early neonatal death |  |  |  |  |  |  |  |  |  |  |
| 2007 | 24 | 11.8 | 19 | 8.4 | 19 | 9.8 | 10 | 8.0 | 14 | 12.8 |
| 2008 | 33 | 14.6 | 44 | 17.4 | 37 | 15.9 | 17 | 12.3 | 23 | 21.3 |
| 2009 | 12 | 5.3 | 49 | 19.6 | 20 | 8.7 | 19 | 14.7 | 22 | 18.5 |
| 2010 | 29 | 9.6 | 49 | 18.1 | 24 | 8.4 | 20 | 13.6 | 34 | 24.5 |
| 2011 | 42 | 12.5 | 42 | 14.9 | 46 | 14.5 | 14 | 8.8 | 24 | 16.7 |
| 2012 | 42 | 10.5 | 42 | 13.5 | 41 | 11.2 | 25 | 13.5 | 18 | 11.3 |
| 2013 | 42 | 9.6 | 41 | 12.0 | 43 | 10.7 | 22 | 10.9 | 18 | 10.4 |
| 2014 | 36 | 7.2 | 43 | 12.9 | 41 | 9.1 | 18 | 8.8 | 20 | 11.2 |
| Neonatal mortality |  |  |  |  |  |  |  |  |  |  |
| 2007 | 29 | 14.2 | 21 | 9.3 | 23 | 11.8 | 12 | 9.6 | 15 | 13.7 |
| 2008 | 44 | 19.5 | 61 | 24.1 | 50 | 21.4 | 20 | 14.4 | 35 | 32.5 |
| 2009 | 20 | 8.8 | 59 | 23.6 | 27 | 11.7 | 23 | 17.8 | 29 | 24.4 |
| 2010 | 35 | 11.6 | 64 | 23.7 | 35 | 12.2 | 25 | 17.0 | 39 | 28.1 |
| 2011 | 52 | 15.5 | 58 | 20.5 | 56 | 17.7 | 23 | 14.5 | 31 | 21.6 |
| 2012 | 52 | 13.0 | 52 | 16.7 | 48 | 13.1 | 32 | 17.2 | 24 | 15.1 |
| 2013 | 55 | 12.6 | 52 | 15.3 | 53 | 13.2 | 31 | 15.3 | 23 | 13.3 |
| 2014 | 47 | 9.4 | 58 | 17.4 | 55 | 12.1 | 23 | 11.2 | 27 | 15.2 |
