# Supplementary figures and images for: Temporal trends in the birth rates and perinatal mortality of twins: A population-based study in China
Source: PLoS One. 2019 Jan 16;14(1):e0209962. doi: 10.1371/journal.pone.0209962 (PMC6334899; doi:10.1371/journal.pone.0209962)

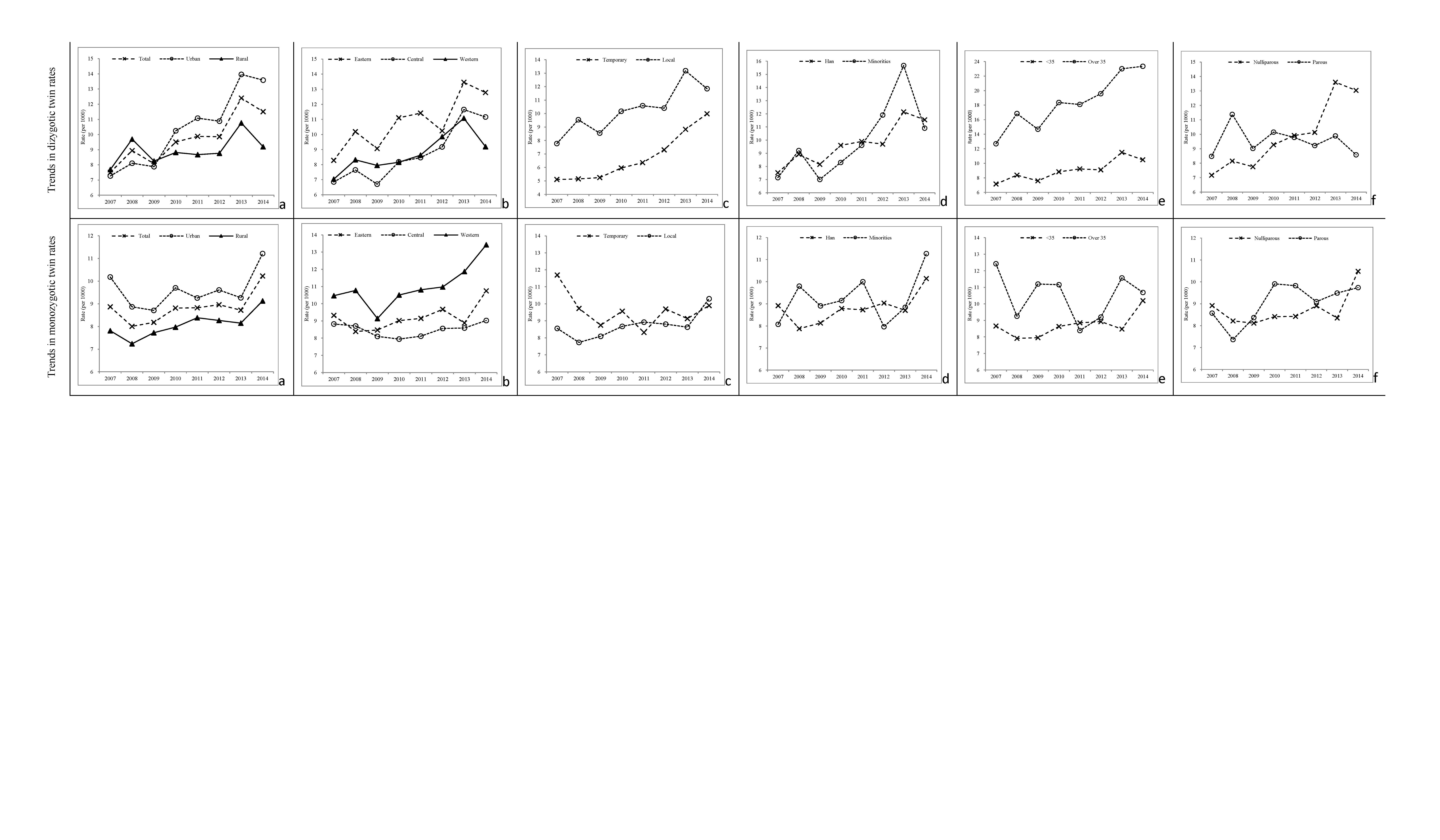

Supplement: S1 Fig — Stratified by: a) urban-rural classification, b) geographic region, c) maternal residence registration, d) maternal ethnicity, e) maternal age, and f) parity. (TIFF) [file pone.0209962.s007.tiff]
